# Experimental investigation of hydrogen production performance of PEM electrolyze

**DOI:** 10.1038/s41598-025-06351-9

**Published:** 2025-07-02

**Authors:** Chen Aijun, Pan Jun, Zhang Hang, Feng Keying, Liu Xubin

**Affiliations:** 1 Guangzhou Power Supply Bureau Electric Power Research Institute, Guangzhou, 510420 China; 2 Sichuan Energy Internet Research Institute, Chengdu, 610500 China

**Keywords:** PEM electrolyze, Hydrogen production, Cold start-up, Shutdown, Thermal start-up, Hydrogen energy, Energy science and technology

## Abstract

As global awareness of environmental protection increases, hydrogen is seen as a promising solution due to its high energy density and zero-emission combustion. The PEM electrolyze combined with renewable energy power generation is an effective method to solve the problem of hydrogen production. The market competitiveness of PEM electrolyte will be enhanced in the future, and the equipment cost can be reduced by 35.8%. The fast dynamic response performance of PEM electrolyzes, especially during start-up and shutdown, affects system flexibility and stability. The 190 Nm^3^/h test platform is established to study the fast dynamic response performance, considering the cold start-up, thermal start-up and shutdown behaviors. The results shown that the 190 Nm³/h PEM electrolyze required 6340 s to achieve cold start-up, 1100 s to achieve thermal start-up, and 855 s to complete shutdown. When operating stably, the temperature fluctuation of the PEM remains below 5 °C, demonstrating the excellent temperature control performance. However, during cold start-up and shutdown, the concentrations of hydrogen and oxygen fluctuate significantly which can easily lead to a decrease in system performance. These findings provide guidance for optimizing the design and operating parameters of PEM Electrolyze systems.

## Introduction

As global focus on environmental protection and sustainable development intensifies, clean energy has emerged as the core of the future energy transition^1^. Hydrogen has the characteristics of high energy density and zero pollution^2^. It is widely regarded as one of the most promising and ideal clean energy sources^3^. There are various ways to produce hydrogen, among which the use of renewable energy coupled with electrolysis of water to produce hydrogen is regarded as the most environmentally friendly approach^4,5^. Hydrogen produced in this way is called green hydrogen. At present, there are several technologies for hydrogen production through water electrolysis: alkaline water electrolysis (ALK)^6^, proton exchange membrane electrolysis (PEM)^7^, high-temperature solid oxide electrolysis (SOEC)^8^, and solid polymer anion exchange membrane electrolysis (AEM). PEM can quickly adapt to power fluctuations and is considered highly suitable for coupling with renewable energy for hydrogen production^9–11^. PEM electrolyze for hydrogen production is an advanced technology that relies on proton exchange membranes to facilitate the electrolysis of water. The PEM electrolyze is a key component of the water electrolysis system^12^. It takes pure water as the reactant. The hydrogen ions (protons) produced at the anode are effectively transferred to the cathode by using a proton exchange membrane, and hydrogen gas is produced at the cathode^13^.

Zhang et al.^14^ developed a high-temperature polymer electrolyte membrane and observed that the three-membrane electrode short stack had excellent start-stop performance. Bornemann et al.^15^ reduced energy costs by 4–7% through the optimization of operating parameters for the electrolyze. Xu et al.^16^ created a full-scale PEM Electrolyze model and studied the parameter distribution within the reactor, suggesting that the inlet temperature of the electrolyte be kept below 60 °C. Shakhshir et al.^17^ explored the effects of different assembly methods on the uniformity of current distribution in PEM electrolyzes, concluding that pneumatic devices used for stack assembly resulted in more uniform local current distribution. Karthikeyan et al.^18^ conducted a lifecycle economic analysis of a solar-PEM Electrolyze hydrogen production system, determining the full-cycle Levelized Cost of Hydrogen (LCOH) to range from 17.48 to 24.33 €/kg. Pfennig et al.^19^ developed thermodynamic and chemical models for PEM electrolyzes at the megawatt scale. Tang et al.^20^ used a numerical model to study water transfer and electrochemical reactions in the PEM porous catalyst layer. Zhou et al.^21^ and Liu et al.^22^ designed a novel flow channel based on a two-phase flow PEM electrolyze model with various flow channel configurations to balance current density and product concentration. Crespi et al.^23^ carried out dynamic modeling of a commercial 60 kW PEM electrolyze system to explore parameter control strategies under various operating conditions. Villagra et al.^10^ investigated the impact of high current density on electrolyze performance and presented the current-voltage polarization curve at a current density of 10 A/cm^2^. Erman et al.^24^ emphasized the importance of temperature and pressure management methods for system reliability and efficiency.

The fast dynamic response performance of the PEM directly affects the dynamic response performance of the system. However, considering factors such as equipment cost and safety issues, there are relatively few experimental studies on PEM electrolyzes. To evaluate the fast dynamic response performance intuitively, the cost change trend of PEM electrolyzes is studied in this manuscript. And established an electrolysis test bench of 190 Nm^3^/h. Experiments on cold start-up, thermal start-up and shutdown behaviors are systematically carried out to study the fast dynamic response performance of the system. Provide specifications for the optimal design and operation parameter determination of large-scale PEM.

## Technical analysis

### Operational principle

**Fig. 1:**
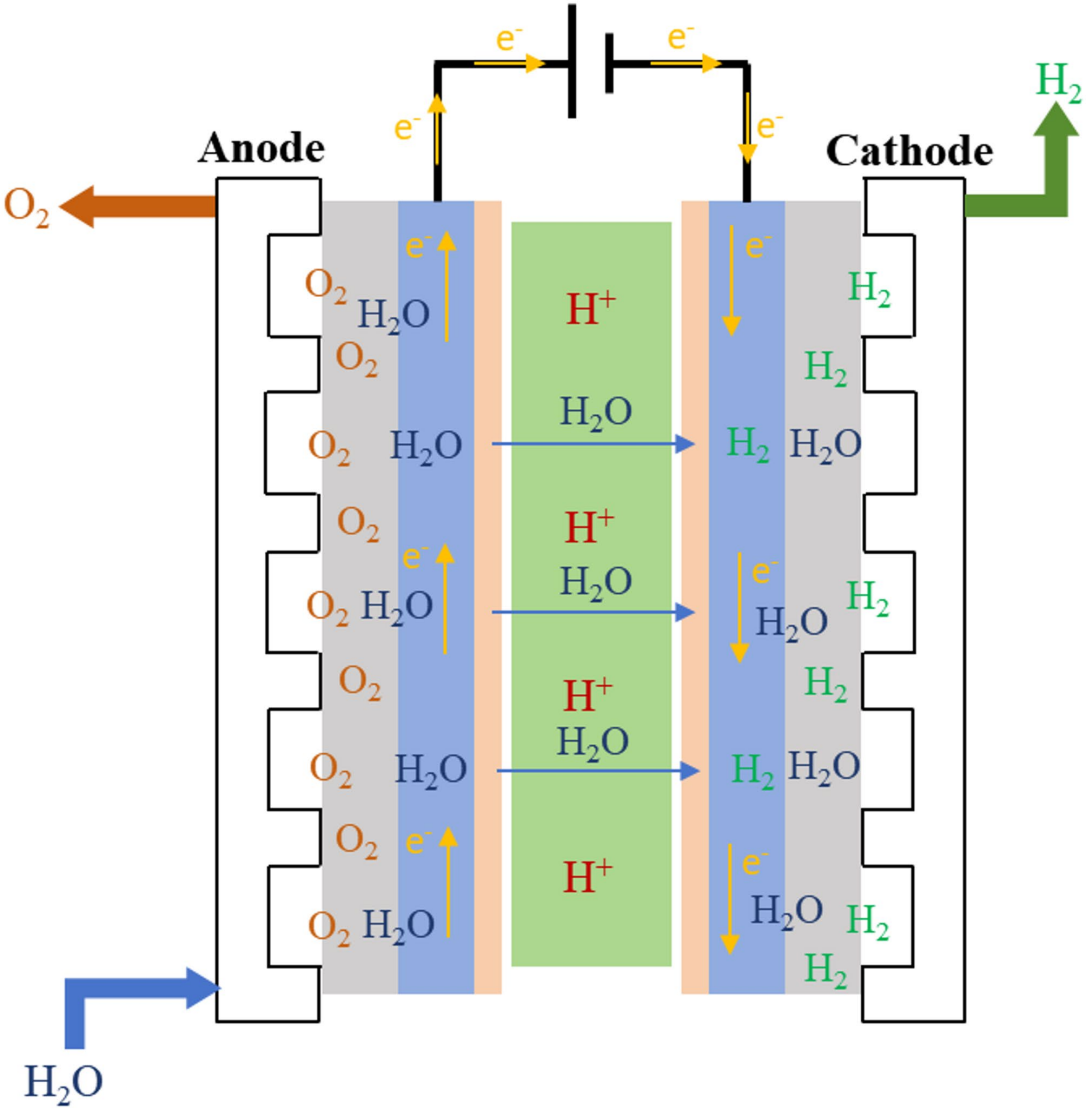
Schematic of PEM electrolyzes.

PEM Electrolyzes of water to produce hydrogen is a process that uses electrolysis to decompose water into hydrogen and oxygen. As shown in Fig. 1, the PEM Electrolyzes has a cathode and an anode. At the anode, water molecules lose electrons (oxidation reaction), forming oxygen and protons. At the cathode, H^+^ migrates to the cathode through PEM and then combines with electrons to form hydrogen gas. The parameters of PEM electrolyzes are listed in the Table 1. The cell electrolytic efficiency of PEM based on LHV is 70–90%.

**Table 1:** Parameters of PEM electrolyzes.

Name	PEM
Temperature (°C)	70–80
Cell efficiency (%)	70–90
Energy consumption (kWh/Nm^3^)	3.8–5
Start-up times (s)	1200–1800

### Cost forecasting

The hydrogen production cost of PEM electrolyzes directly affects their commercial development. Table 2 presents the boundary conditions for calculating the cost of hydrogen production by PEM electrolyzes. The cost of hydrogen production includes equipment investment, labor and auxiliary material expenses, among which equipment investment is the main cost driver. Under typical industrial electricity consumption conditions in China, the electricity price is 0.066 USD/kWh, the equipment investment is 4,120 USD NM^3^/h, and the hydrogen production cost is 0.531 USD/Nm^3^.

**Table 2:** The boundary for the cost of hydrogen production by PEM electrolyzes^25^.

Name		Parameter
Depreciation ratio	–	The 10-year linear depreciation is 0
Raw material costs	USD/kWh	0.066
Investment of equipment	USD NM^3^/h	4120
O&M/investment ratio	%	3
Raw material consumption	kWh/Nm^3^	4.5
Cost of hydrogen production	USD/Nm^3^	0.531

**Fig. 2:**
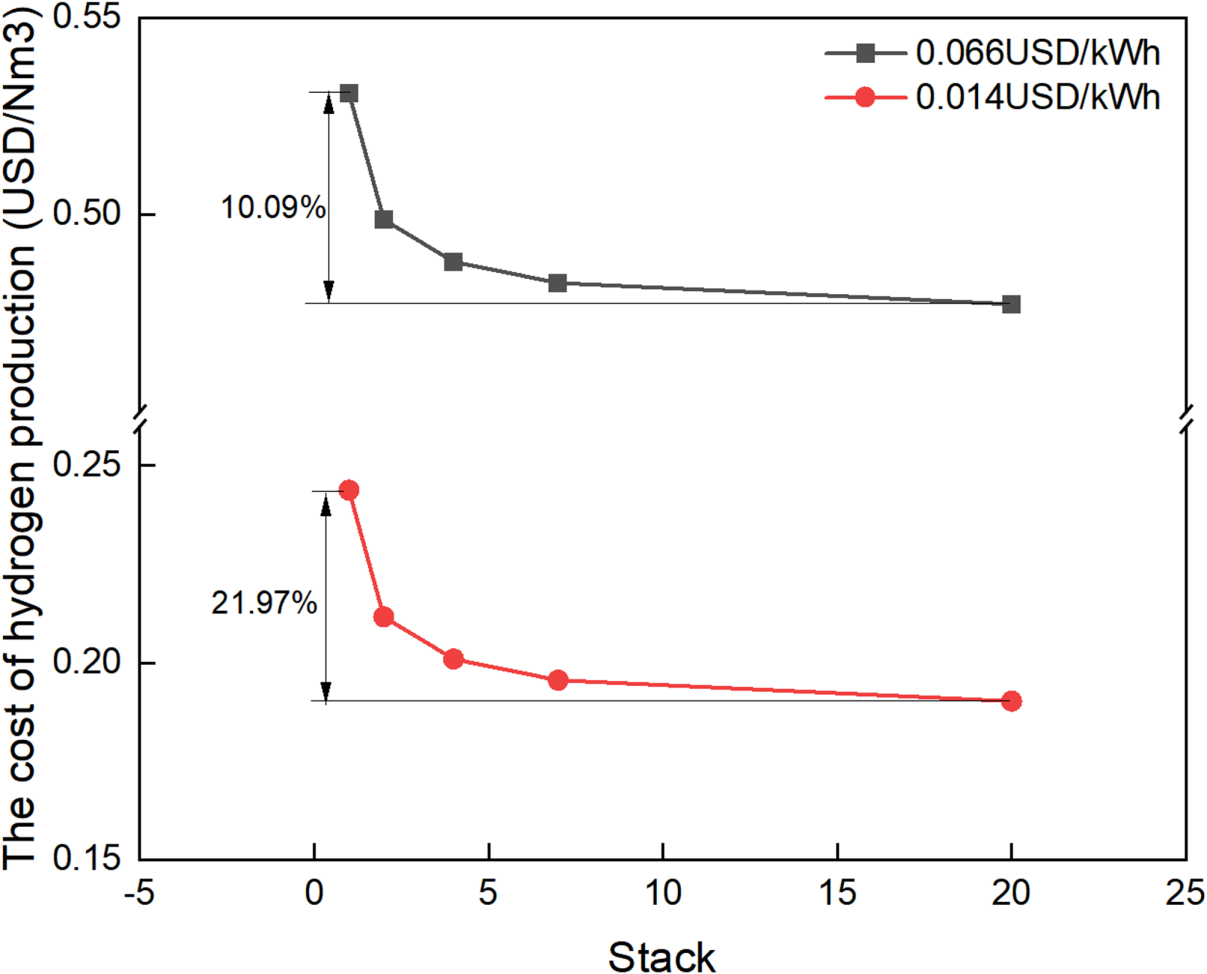
The trend of cost changes in PEM electrolyzes.

With the development of large-scale PEM electrolyzes, water electrolysis technology is advancing rapidly and is expected to significantly reduce capital expenditure. It is expected that the cost of 6-stack PEM electrolyzes will be reduced by 40%, and the electrolyze stack accounts for 60% of the capital expenditure on PEM electrolyze technology^24^. Meanwhile, PEM electrolyzes can be coupled with renewable energy power generation to produce hydrogen using off-peak electricity. As shown in Fig. 2, under both industrial power consumption (0.066 USD/kWh) and renewable energy power generation (0.014 USD/kWh) conditions, the hydrogen production cost of PEM electrolyzes decreases with the increase in the number of stacks. Hydrogen production using renewable energy can reduce the cost of hydrogen production by approximately 21.97%. In the future, the cost of electrolyzes will further decrease. Meanwhile, when coupled with renewable energy power generation, the cost of hydrogen production will only be 35.8% of the current level. When the number of stacks is small, the cost of electrolyzes has a significant impact on the cost of hydrogen production. When the number of stacks increases to a certain amount, due to the boundary effect, the sensitivity of the cost of electrolyzes to the cost of hydrogen production decreases.

## Experiment of PEM electrolyze

### Experiment bench

**Fig. 3:**
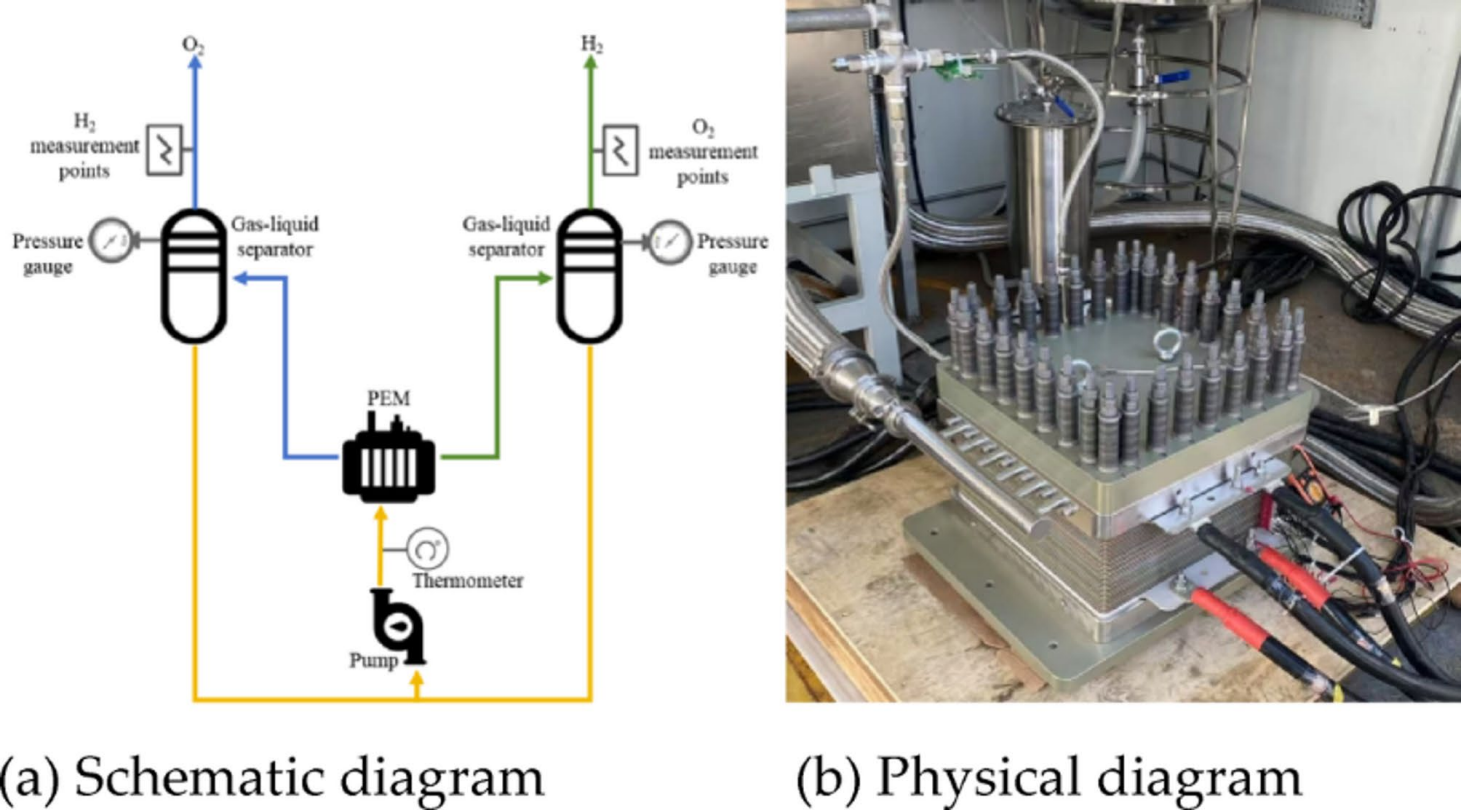
The PEM electrolyze experimental platform.

The fast dynamic response performance of hydrogen production by PEM electrolyzes is studied, and a 190 Nm^2^/h PEM electrolyze experimental platform is established, as shown in Fig. 3. The experimental platform is composed of auxiliary equipment such as PEM electrolyze, hydrogen separator, oxygen separator, pressure gauge, thermometer and pump. The hydrogen and oxygen produced by the PEM electrolyze are mixed with the electrolyte and then separated by a hydrogen and oxygen separator. The separated electrolyte is recirculated back into the PEM electrolyte by a pump. Due to incomplete gas-liquid separation, gas crossover phenomena will occur in the PEM electrolysis system^26^. Therefore, to ensure the safe operation of the system, oxygen purity analyzers for hydrogen and hydrogen purity analyzers for oxygen are typically installed in water electrolysis hydrogen production devices. The PEM electrolyze operating parameters is shown in Table 3. The time of start-up is the time required for the current to rise from 0 A to the rated current. The time of shutdown is the time required for the current to decrease from the rated current to 0 A.

**Table 3:** PEM electrolyze operating parameters.

Name	Unit	Index
Cell	–	120
Rated hydrogen production	m^3^/h	190
Current rating	A	3800
Cold start-up temperature	℃	26
Cold start-up pressure	MPa	0.12
Thermal start-up temperature	℃	63.5
Thermal start-up pressure	MPa	1.58

The raw water used in this experiment complies with the relevant provisions of the Chinese national standard GB/T 37562-2019, “Technical Specifications for Pressure-Type Water Electrolysis Hydrogen Production Systems,” as detailed in Table 4.

**Table 4:** Raw water quality parameters.

Name	Unit	Index
Conductivity (25 °C)/resistivity	mS/m Ω·cm	≤ 0.10 ≥ 1.0 × 10^6^
Oxidizable substance concentration (O)	mg/L	≤ 0.08
Absorbance (254 nm, 1 cm pathlength)	–	≤ 0.01
Evaporation residue (105 °C ± 2 °C)	mg/L	≤ 1.0
Soluble silicon (as SiO_2_)	mg/L	≤ 0.02

### Experimental methods

The startup and shutdown process of the PEM electrolyze includes three distinct phases: cold start, thermal start-up, and shutdown. For experimental convenience, the cold start-up is conducted first, followed by the shutdown test, and finally, the thermal start-up test. The experimental procedure is outlined as follows:

1. Before initiating the cold start trial, the hydrogen production system is kept in a shutdown state for 3 h, maintained at a temperature of 26 °C and a pressure of 0.12 MPa.
2. During the cold start test, power to the hydrogen production system is activated, and the oxygen-hydrogen instrument is preheated. The input current to the electrolyze is progressively increased via the control software interface until the rated current (3800 A) is reached, which is then maintained for 15 min to complete the cold start process.
3. Prior to the shutdown test, the hydrogen production system operates stably for 20 min at the rated power point, with a constant current of 3800 A.
4. In the shutdown test, the input current to the electrolyze is gradually reduced via the control software interface, following the prescribed procedure, until the current reaches zero, thereby completing the shutdown process.
5. Before beginning the thermal start-up test, the hydrogen production system is held in a shutdown state for 1 min, maintained at a temperature of 63.5 °C and a pressure of 1.58 MPa.

During the experiment, the time taken for the hydrogen production system to reach the rated current (3800 A) from the moment power is supplied is recorded. Additionally, the time required for the oxygen concentration in hydrogen (O_2_ in H_2_) and the hydrogen concentration in oxygen (H_2_ in O_2_) to fall within the acceptable limits is recorded. The target concentrations are O_2_ in H_2_ ≤ 0.5% and H_2_ in O_2_ ≤ 2%. To ensure precise tracking of the system’s performance, data for DC voltage, current, temperature, pressure, O_2_ in H_2_, and H_2_ in O_2_ are measured at 1-s intervals.

## Hydrogen production performance

### Cold start-up

**Fig. 4:**
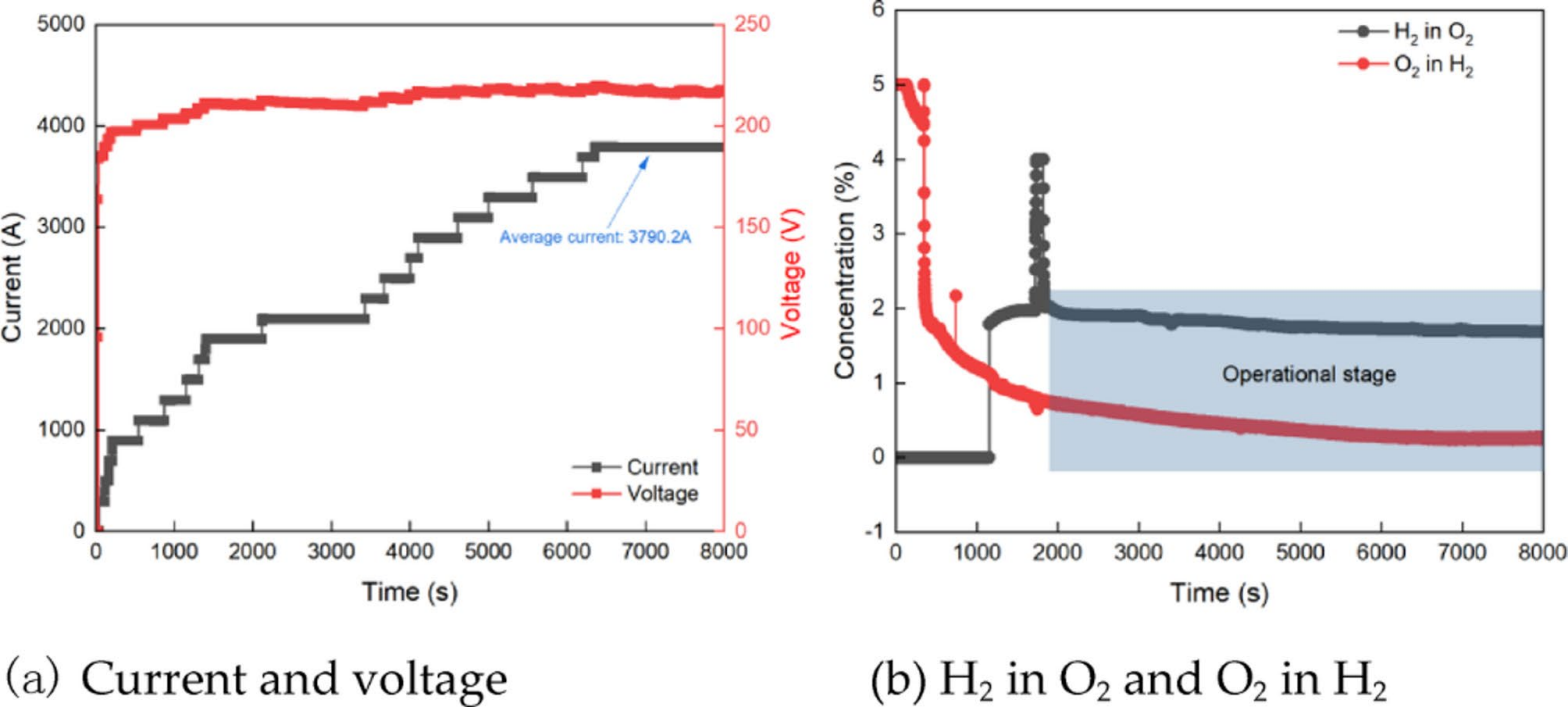
Operating performance during cold start-up.

Figure 4a presents the current and voltage curves during the cold start-up. Within the first 24 s after the system is powered on, the voltage increases rapidly, reaching 182 V at 32 s. Subsequently, it gradually rises to approximately 215 V. As time progresses, the current increases steadily, and by around 6340 s, the rated current is achieved, marking the completion of the cold start. Figure 4b illustrates the changes in the oxygen concentration in hydrogen (O_2_ in H_2_) and hydrogen concentration in oxygen (H_2_ in O_2_) in the separator during the cold start-up of the PEM electrolyze. After the PEM electrolyze is started, the oxygen concentration at the hydrogen outlet of the hydrogen separator gradually decreases. A sharp drop in oxygen concentration occurs between 340 and 400 s, and then continued to decline slowly. After 6340 s, when the rated current is reached, the O_2_ in H_2_ concentration stabilizes at approximately 0.25%. The H_2_ in O_2_ detector, located in the oxygen separator, requires 30 min of preheating before it becomes operational. Once the preheating process is completed, the H_2_ in O_2_ concentration gradually decreases. By the time the cold start-up is fully completed, the H_2_ in O_2_ concentration is approximately 1.69%.

**Fig. 5:**
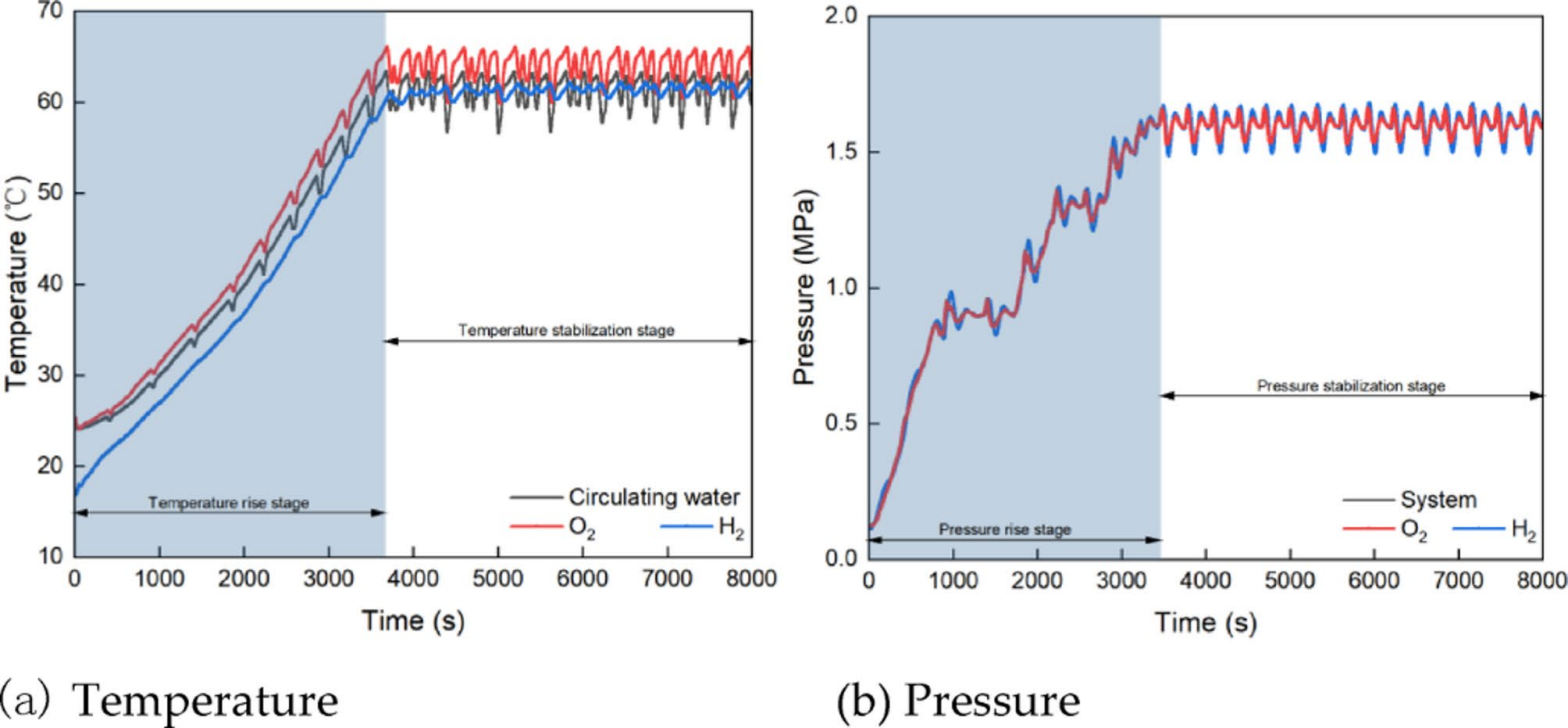
Temperature and pressure curves during cold start up.

Figure 5a shows the temperature variations of circulating water, hydrogen, and oxygen during the cold start phase of the experiment. From 0 to 3670 s after starting, the temperatures increase gradually from room temperature to 63 °C, 65 °C, and 60 °C, respectively. During the start-up from 3671s to 8000 s, the temperature fluctuated within a range of approximately 1–2 °C. These fluctuations are relatively minor, indicating stable temperature control within the system. The relatively stable temperatures at each point within the electrolyte system indicate that the PEM electrolytic cell has excellent cold start performance. Figure 5b illustrates the pressure variation during the cold start-up. The pressure increases gradually after startup, following a similar trend to the temperature. At 3670 s, the system pressure, hydrogen pressure, and oxygen pressure stabilize between 1.4 and 1.6 MPa, with steady fluctuations. This steady fluctuation indicates stable pressure within the system during operation.

### Shutdown stage

**Fig. 6:**
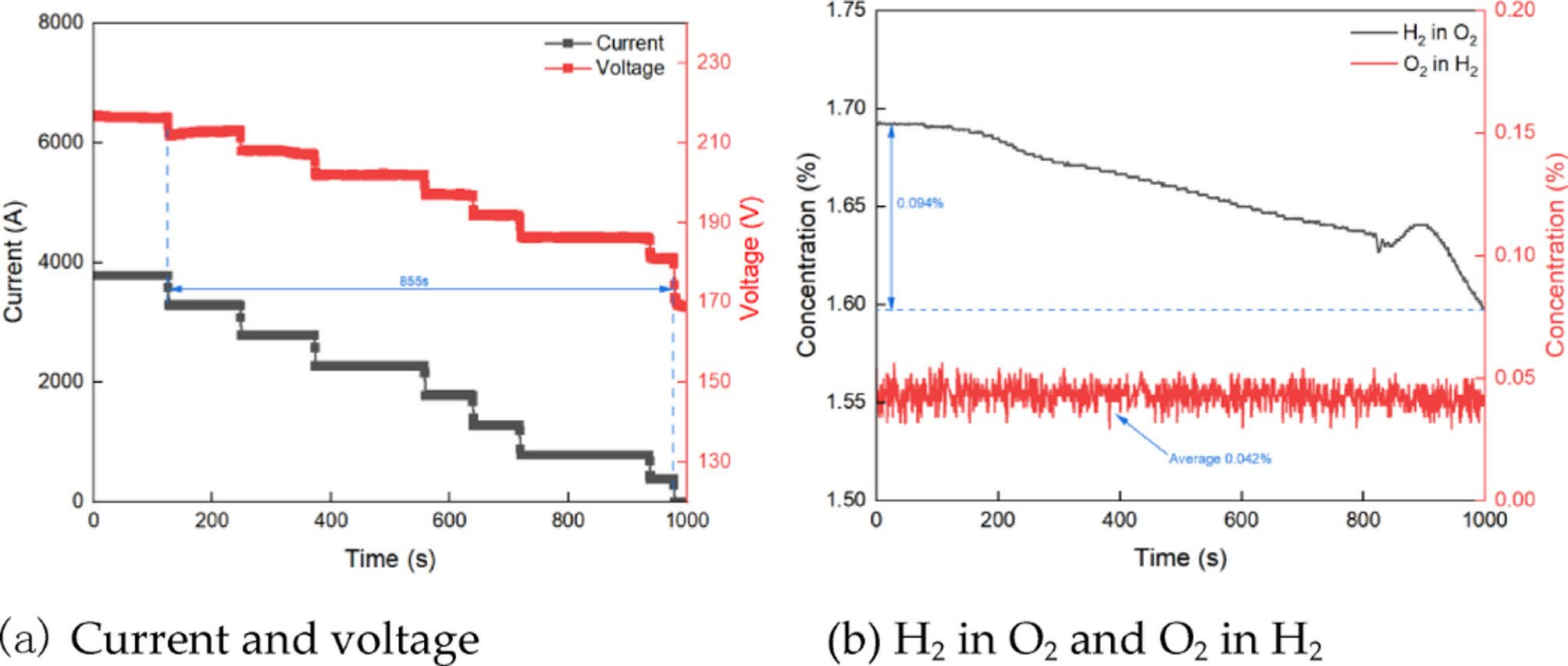
Operating performance during the shutdown.

Figure 6a shows the current and voltage variation during the system shutdown. Once the shutdown process begins, both the current and voltage decrease gradually. At approximately 855 s, the input current to the electrolyze reaches 0 A for the first time, signaling the completion of the shutdown process. Figure 6b depicts the variations in O_2_ in H_2_ and H_2_ in O_2_ concentrations at the outlet of the separators during the shutdown. Once the PEM electrolyze starts the shutdown, the O_2_ in H_2_ fluctuates, with an average value of about 0.042%. The H_2_ in O_2_ decreases gradually as the shutdown time increases. Around 810 s, the H_2_ in O_2_ first increases, then decreases again, eventually reaching a final value of 0.094%. This phenomenon can likely be attributed to the gradual reduction of hydrogen in the oxygen separator during the shutdown. As the hydrogen density decreases, its concentration drops, causing hydrogen to accumulate locally in the separator. As H_2_ gradually accumulates, when it reaches the critical level, H_2_ and O_2_ begin to separate, and then H_2_ in O_2_ decrease.

### Thermal start-up

**Fig. 7:**
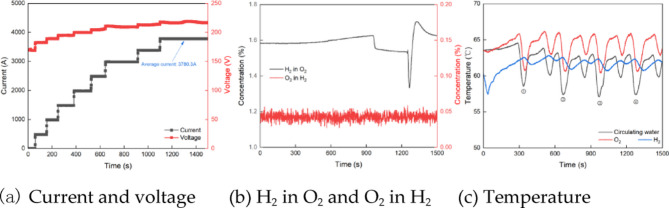
Operating performance during the thermal start-up.

Figure 7a shows the changes of current and voltage during the thermal start-up process. As the PEM electrolyze starts up, both the current and the voltage increase steadily. After approximately 1100 s, the average current reached 3780.3 A, indicating that the startup process is completed. Compared with Fig. 4, the time of thermal start-up is shortened by 520 s, which is only 17.35% of the cold start-up. Furthermore, during the thermal start-up, the increase in current and voltage is more consistent. Compared with the cold start-up, the changes of current and voltage during the thermal start-up are more stable and regular.

Figure 7b presents the change curves for H_2_ in O_2_ and O_2_ in H_2_ concentrations during the thermal start-up. As the start-up time increases, the O_2_ in H_2_ fluctuates within a narrow range, with an average value of approximately 0.040%. This is comparable to the O_2_ in H_2_ observed during cold start-up. With the increase of time, the concentration of H_2_ in O_2_ increases. After 900s of thermal start-up, the concentration of H_2_ in O_2_ decreased briefly. However, it rebounded to the peak at 1200 s and then gradually stabilized. The reason for this phenomenon is that the hydrogen concentration detector at the outlet of the oxygen separator is preheated for 900 s. Figure 7c shows the temperature variation curve during the thermal start-up. As the start-up proceeds, the temperatures of the circulating water, oxygen and hydrogen experience periodic fluctuations. At 1100 s, the thermal start-up is completed. The PEM electrolyze experienced three different temperature fluctuations during the thermal start-up, which were marked as ① to ③ respectively. Despite these fluctuations, the system remained in a relatively stable state over time. It can be seen from the comparison with Fig. 6 that the temperature range reached after cold start-up and thermal start-up is similar, and the fluctuation frequency is also similar.

## Conclusion

With the large-scale production of PEM electrolyzes in the future and the wide application of renewable energy power generation, the cost of hydrogen production by PEM electrolyzes coupled with renewable energy power generation technology is expected to be significantly reduced. It is expected that the production cost will drop to the current 35.8%. The market competitiveness has been significantly enhanced.

The time of cold start-up for the 190 Nm³/h PEM electrolyze is 6340 s, the shutdown is 855 s, and the thermal start-up is 1100 s, with good fast dynamic response performance demonstrated. Once the electrolyze has completed the start-up, periodic fluctuations in the temperatures of the circulating water, hydrogen, and oxygen are observed during the stable operation phase, with an amplitude of less than 5 °C. It indicates that the temperature control performance is good. The O_2_ in H_2_ is approximately 0.25%, and the H_2_ in O_2_ is around 1.69%. To further reduce gas crossover phenomena and improve hydrogen production efficiency, it is recommended that the separation efficiency of the gas-liquid separator in the system be enhanced.

During the shutdown and cold start-up, a sudden decrease and increase in the H₂ in O₂ are observed. It is mainly caused by the preheating of the instrument. Therefore, it is recommended that in actual operation, considering the preheating of the equipment, the start-up time be extended by at least 400s to ensure that the PEM electrolyze meets the set operating conditions.

## Data Availability

All data generated or analysed during this study are included in this published article.
